# STAT3 and STAT5A are potential therapeutic targets in castration-resistant prostate cancer

**DOI:** 10.18632/oncotarget.20844

**Published:** 2017-09-12

**Authors:** Sambit K. Mohanty, Kader Yagiz, Dinesh Pradhan, Daniel J. Luthringer, Mahul B. Amin, Serhan Alkan, Bekir Cinar

**Affiliations:** ^1^ Department of Pathology and Laboratory Medicine, Cedars-Sinai Medical Center, Los Angeles, CA 90048, USA; ^2^ Department of Hematology and Oncology, Cedars-Sinai Medical Center, Los Angeles, CA 90048, USA; ^3^ University of Pittsburgh Medical Center, Pittsburgh, PA 15238, USA; ^4^ Department of Pathology and Laboratory Medicine, University of Tennessee Health Sciences Center, Memphis, TN 38163, USA; ^5^ Department of Biological Sciences, The Center for Cancer Research and Therapeutic Development, Clark Atlanta University, Atlanta, GA 30314, USA; ^6^ Winship Cancer Institute, Emory University, Atlanta, GA 30322, USA

**Keywords:** STAT3, STAT5A, pimozide, castration-resistance, prostate cancer

## Abstract

Mechanisms of castration-resistant prostate cancer (CRPC) are not well understood, thus hindering rational-based drug design. Activation of STAT3/5A, key components of the JAK/STAT pathway, is implicated in aggressive PC, yet their clinical relevance in CRPC remains elusive. Here, we evaluated the possible role of STAT3/5A in CRPC using immunological, quantitative mRNA expression profiling, and pharmacological methods. We observed a strong nuclear immunoreactivity for STAT3 and STAT5A in 93% (n=14/15) and 80% (n=12/15) of CRPC cases, respectively, compared with benign prostatic hyperplasia (BPH). We demonstrated that PC cells express varying levels of STAT3 and STAT5A transcripts. In addition, we demonstrate that pimozide, a psychotropic drug and an indirect inhibitor of STAT5, attenuated PC cells growth, and induced apoptosis in a dose-dependent manner. Furthermore, our analysis of the PC public data revealed that the STAT3/5A genes were frequently amplified in metastatic CRPC. These findings suggest that STAT3/5A potentially serves as a predictive biomarker to evaluate the therapeutic efficacy of a cancer drug targeting the JAK/STAT pathway. Since the JAK/STAT and AR pathways are suggested to be functionally synergistic, inhibition of the JAK/STAT signaling alone or together with AR may lead to a novel treatment modality for patients with advanced PC.

## INTRODUCTION

Prostate cancer (PC) is the most frequently diagnosed non-cutaneous malignancy in males in the United States [1]. Androgen hormone/androgen receptor (AR) signaling are central to PC development, progression, and metastasis [2–6]. Therefore, androgen deprivation therapy (ADT) is the first line therapy for advanced PC. However, almost all patients who received ADT develop metastatic castration-resistant prostate cancer (CRPC) [7–13]. In addition, ADT has limited effects on long-term survival and side effects, making it undesirable for PC patients [14]. Recurrent cases are usually treated by cytotoxic chemotherapeutic agents, which have narrow therapeutic indices due to their high cytotoxic effects [13, 15–18]. Unfortunately, patients with metastatic CRPC do not respond well to cytotoxic agents and show high mortality rate. Therefore, finding a suitable target and a therapeutic agent with minimal or insignificant side effects, while also being effective on the recurrent cancer, would have major impact on the management of patients with CRPC.

Signal transducer and activator of transcription 3 (STAT3) and highly homologous isoforms STAT5A and STAT5B (STAT5A/B) are key components of the Janus tyrosine kinase (JAK)/STAT pathway [19]. Both STAT3 and STAT5 act as signaling proteins in the cytoplasm and transcription factors when they localize to the nucleus [19]. The STAT3 and STAT5 are activated by phosphorylation at a specific tyrosine (Y) residue such as phospho-Y705 in STAT3 (pY705-STAT3) and phospho-Y694 in STAT5A (pY694-STAT5A), followed by translocation into the nucleus where they bind *cis-*DNA and activate target genes to exert their biology [20–28]. Several growth factors such as epidermal growth factor receptor (EGFR), hepatocyte growth factor receptor (HGFR), and platelet-derived growth factor receptor (PDGFR) and non-receptor cytoplasmic tyrosine kinases such as Abelson leukemia protein and Src-related kinases have demonstrated to activate STAT3 [20–28]. Evidence suggest that STAT3 and STAT5 play critical roles in cancer progression, thus making themselves as therapeutic targets [29]. Activation of STAT3 (pY705-STAT3) and STAT5 (pY694-STAT5A) has been implicated in treatment-naïve as well as advanced PC [30–38]. Therefore, targeting of STAT3 and STAT5 in PC could in practice be of therapeutic significance. Expression of STAT3 and STAT5 has been demonstrated in PC cell lines [32, 35, 37] and PC tissues [30, 31, 33, 34, 36, 38]; however, the expression of STAT3 and STAT5A in metastatic CRPC has been rarely studied [31, 33, 38].

In this study, we evaluated the expression and possible role of STAT3/5A in CRPC. We observed a strong nuclear immunoreactivity for activated STAT3 (pY7705) and STAT5A (pY694) in CRPC cases compared with BPH. In addition, we found that up to 20% of CRPC clinical cases showed a frequent amplification of the STAT3 and STAT5A genes. Moreover, we showed that PC cells C4-2 and PC3 expressed low levels of STAT3 and STAT5A mRNA and were relatively resistant to pimozide [39] in comparison with 22Rv1 cells that express high levels of STAT3 and STAT5. These findings suggest that STAT3 and STAT5A may serve as a predictive biomarker for evaluating the responsiveness of therapies targeting the JAK/STAT pathway.

## RESULTS

### Demographics, clinical, and histopathologic parameters

CRPC cases used in the current study were diagnosed on transurethral resection of prostate for obstructive uropathy. All patients with CRPC were treated with ADT and developed CRPC. Ages ranged from 59 years to 95 years (median = 81 years and mean = 80.6 years) in the CRPC cases and 57 years to 86 years (median = 68 years and mean = 71 years) in the BPH category. Prostate tumor tissues analyzed were from patients who were treated ADT and developed resistance to it. The staining was performed on the primary tumors obtained by transurethral resection in all cases. Radical prostatectomy was not performed in any of patients with CRPC. 46% of cases (n=7) had metastatic disease. Additional therapies included chemotherapy (3 cases) and radiation therapy (2 cases). 46% (n=7) of cases displayed Gleason patterns 4 (primary) and 5 (secondary), 27% (n=4) of cases showed Gleason patterns 5 (primary) and 4 (secondary), and 27% (n=4) of cases exhibited Gleason patterns 5 (primary) and 5 (secondary). Evidence suggests that major proportions of prostate tumors exhibit characteristics of mixed neuroendocrine (NE) carcinoma-acinar adenocarcinoma, small cell carcinoma, and large cell carcinoma with history of hormonal therapy [40]. Therefore, NE markers performed in these cases were retrieved from the departmental archive and were studied. None of the cases analyzed in this study exhibited NE phenotype (small cell or large cell neuroendocrine carcinoma) and stained for NE markers (synaptophysin, chromogranin, and CD56). As reported previously, all tumors utilized in this study showed multifocal to diffuse and strong nuclear immunoreactivity for androgen receptor and NKX3.1, a homeobox-containing transcription factor that functions as a negative regulator of epithelial cell growth in the prostate [41, 42]. Its aberrant expression is associated with prostate tumor progression. A weak and sporadic expression of prostate specific antigen (PSA) was observed in only three (20%) of the tumors. Four of the metastatic cases were Gleason patterns 4 and 5 (one with 5 and 4, and two with 5 and 5). Three cases with Gleason patterns 4 and 5, and one case each with patterns 4 and 5, and 5 and 5 were treated with radiation therapy. Four cases with Gleason patterns 4 and 5 and one case with Gleason patterns 5 and 4, and 5 and 5 were treated with chemotherapy. The cases that were treated with both chemotherapy and radiation therapy were of Gleason patterns 4 and 5 (Table 1).

**Table 1:** Clinicopathological characteristics and immunoexpression for STAT3 and STAT5A in cohort of CRPC cases

Case	Age (years)	Specimen Type	Gleason Score (Primary+ Secondary pattern)	Prognostic Group Grade	ADT	Radical Prostate-ctomy	CRPC	Metastasis	Radiation Therapy	Chemo-therapy	STAT3 Expression	STAT5A Expression
1	59	TURP	9(4+5)	5	Yes	No	Yes	Yes	Yes	Yes	3+ and strong	3+ and strong
2	69	TURP	9(4+5)	5	Yes	No	Yes	No	No	Yes	3+ and strong	3+ and strong
3	69	TURP	9(5+4)	5	Yes	No	Yes	No	No	No	2+ and strong	2+ and strong
4	70	TURP	10(5+5)	5	Yes	No	Yes	No	No	No	2+ and strong	2+ and strong
5	76	TURP	9(4+5)	5	Yes	No	Yes	Yes	Yes	Yes	3+ and strong	3+ and strong
6	80	TURP	9(5+4)	5	Yes	No	Yes	No	No	No	2+ and strong	2+ and strong
7	81	TURP	9(5+4)	5	Yes	No	Yes	No	No	Yes	2+ and strong	Negative
8	81	TURP	9(4+5)	5	Yes	No	Yes	Yes	No	No	3+ and strong	3+ and strong
9	86	TURP	10(5+5)	5	Yes	No	Yes	No	No	No	Negative	Negative
10	86	TURP	10(5+5)	5	Yes	No	Yes	Yes	Yes	No	3+ and strong	3+ and strong
11	86	TURP	9(5+4)	5	Yes	No	Yes	Yes	Yes	No	2+ and strong	3+ and strong
12	88	TURP	9(4+5)	5	Yes	No	Yes	No	No	No	2+ and strong	Negative
13	91	TURP	9(4+5)	5	Yes	No	Yes	No	No	No	2+ and strong	2+ and strong
14	92	TURP	9(4+5)	5	Yes	No	Yes	Yes	Yes	Yes	2+ and strong	3+ and strong
15	95	TURP	10(5+5)	5	Yes	No	Yes	Yes	No	Yes	3+ and strong	3+ and strong

Abbreviations: TURP: transurethral resection of prostate gland. ADT: Androgen deprivation therapy; CRPC: Castration-resistant prostate cancer

### CRPC tissues exhibit strong nuclear immunoreactivity for STAT3 and STAT5A

**Figure 1:**
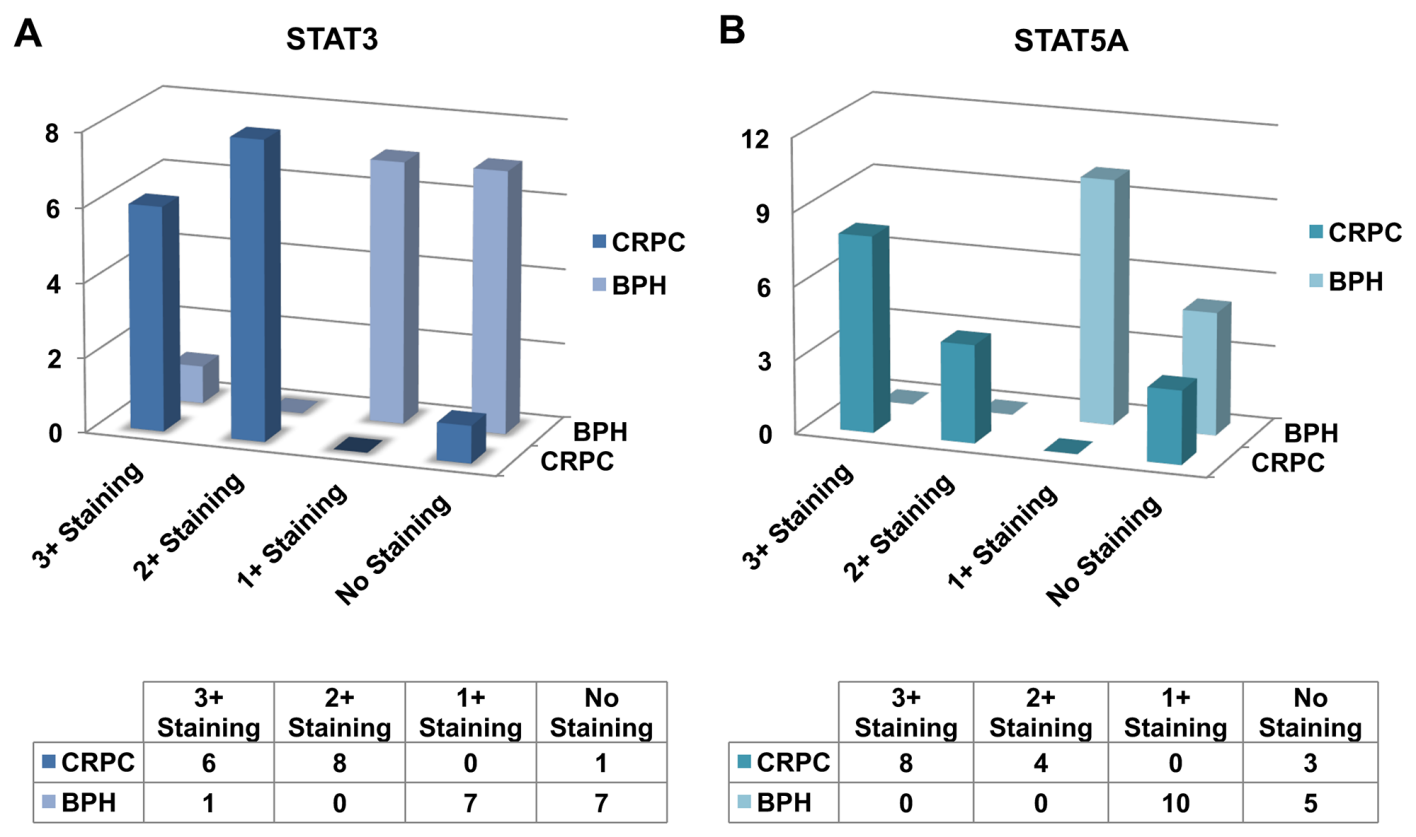
Bar diagram showing the expression patterns of STAT3 **(A)** and STAT5A **(B)** in castration-resistant prostate cancer (CRPC) and benign prostatic hyperplasia (BPH) clinical cases as analyzed by IHC.

**Figure 2:**
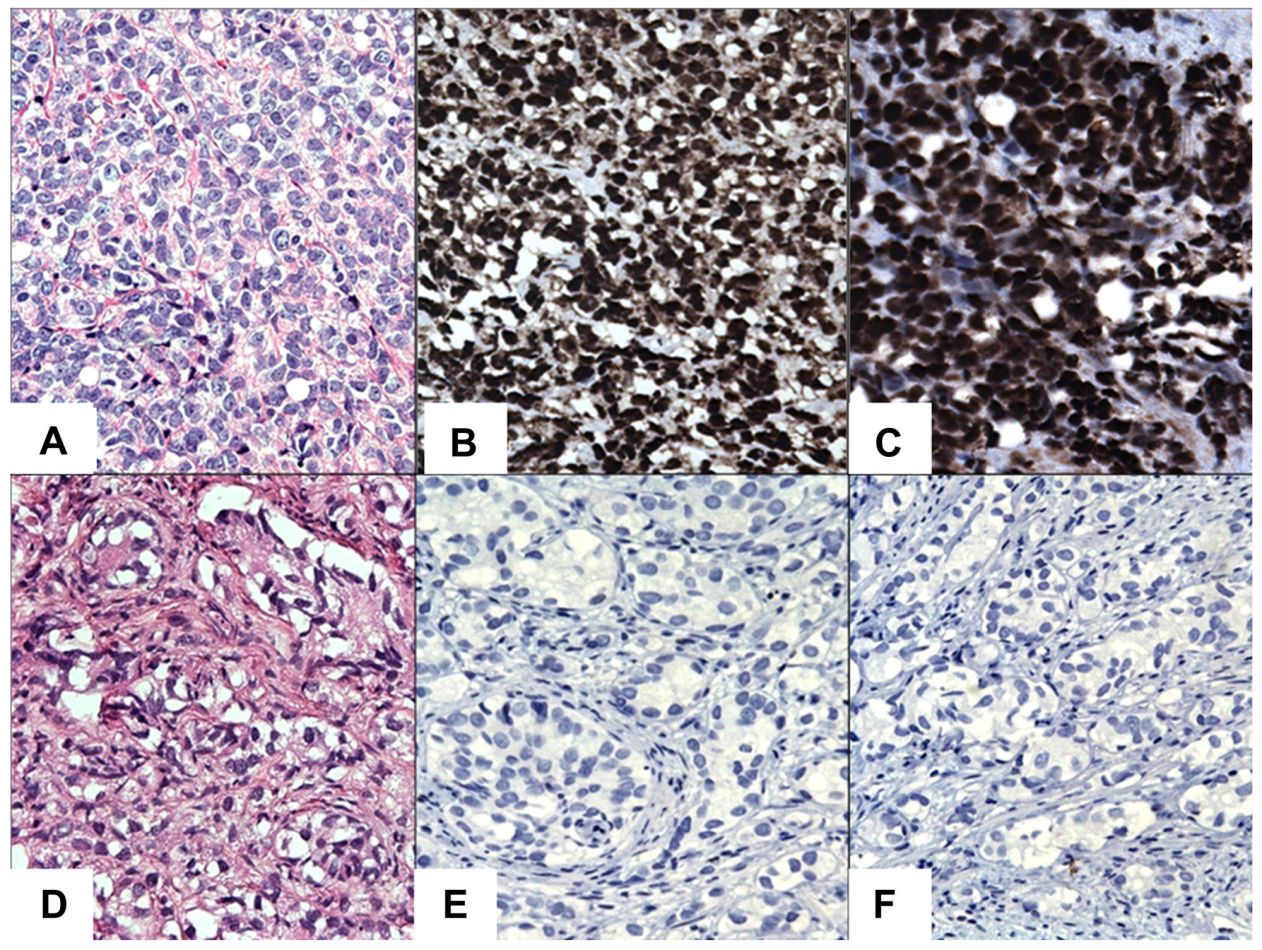
IHC staining of STAT3/5A in CRPC cases **(A)** Hematoxylin and Eosin (H&E). **(B)** Diffuse and strong nuclear immunoreactivity for STAT3. **(C)** Diffuse and strong nuclear immunoreactivity for STAT5A. **(D)** A case of H&E staining in CRPC. **(E)** Negative staining for STAT3 in CRPC. **(F)** Negative staining for STAT5A in CRPC. Original magnification was x200. Micrographs are representation of multiple images.

**Figure 3:**
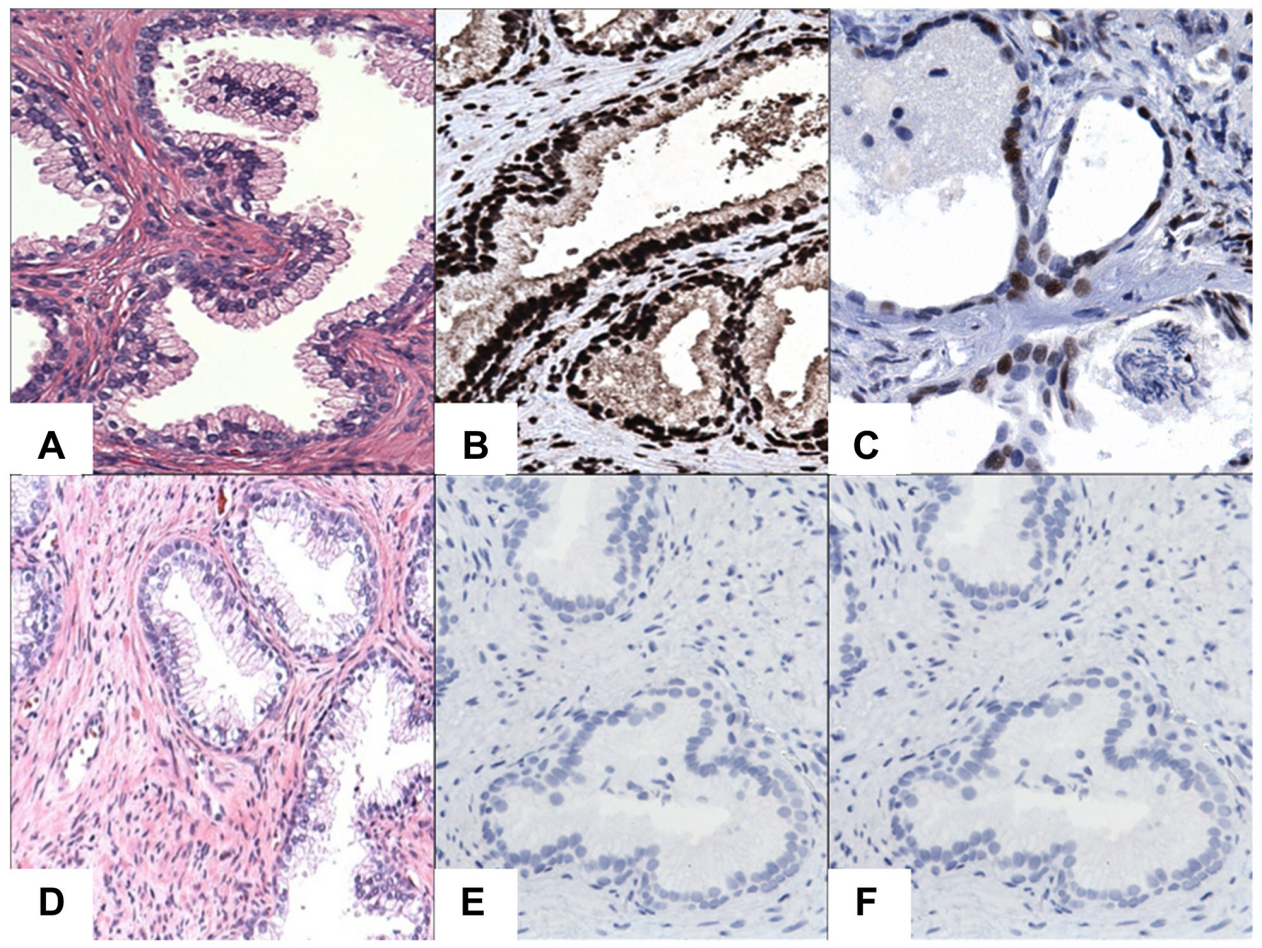
IHC staining of STAT3/5A in BPH cases **(A)** Hematoxylin and Eosin (H&E) staining of BPH cases. **(B)** Diffuse and strong nuclear immunoreactivity for STAT3. **(C)** Diffuse and moderate to strong nuclear immunoreactivity for STAT5A. **(D)** A case of H&E staining in BPH. **(E)** Negative staining for STAT3 in BPH. **(F)** Negative staining for STAT5A in BPH. Original magnification was x200. Micrographs are representation of multiple images.

To gain insight into the role of STAT3 and STAT5A in advanced PC, we evaluated the patterns of STAT3 and STAT5A protein expression in a cohort of CRPC (n=15) and BPH (n=15) cases by IHC (Figure 1, 2, and 3). As outlined in Table 2, pSTAT3 (Y705) and pSTAT5A (Y694) antibodies were used in IHC to assess the levels of activated, nuclear STAT3 and STAT5A proteins. Strong nuclear immunoreactivity for STAT3 and STAT5A were observed in 93% (n=14) and 80% (n=12) of CRPC cases, respectively, whereas only one (7%) BPH case showed strong STAT3 staining (Figure 1A, 1B, respectively). Focal and weak positivity for STAT3 and STAT5A were noted in 47% (n=7) and 67% (n=10) of BPH cases. None of the BPH cases showed strong STAT5A expression. Of 14 cases, which showed strong immunoreactivity for STAT3 in CRPC cases, diffuse staining pattern was observed in 6 cases and multifocal staining was observed in 8 cases (Figure 1A; Figure 2B, 2C). Diffuse and strong STAT5A expression was evident in 8 and 4 CRPC cases, respectively (Figure 1B; Figure 3B, 3C). None of CRPC cases showed weak staining. In contrast, 7 of 10 cases of BPH showed focal and weak staining for STAT3 (Figure 1A). Only one BPH case showed strong and diffuse staining for STAT3 (Figure 1A) and none showed strong STAT5A expression (Figure 1B). All metastatic cases were STAT3 (multifocal and strong, n=2 and diffuse and strong, n=5) and STAT5A positive (diffuse and strong) (Figure 2; Figure 3, respectively). The cases that were treated with both chemotherapy and radiation therapy exhibited diffuse and strong immunoreactivity for STAT5A in 3 cases and diffuse and strong and multifocal and strong immunoreactivity for STAT3 in 2 and 1 cases, respectively. All the cases treated with chemotherapy alone depicted diffuse and strong reaction for STAT3 (n=3) and diffuse or multifocal and strong (n=2) and no (n=1) reaction for STAT5A, respectively. One of the cancer cases treated with only radiation therapy showed diffuse and multifocal positivity for STAT3 and 2 cases that were treated with radiation therapy were diffuse and strongly positive for STAT5A (Table 1 to 5). The cases with weak and patchy or focal positivity for PSA exhibited diffuse and strong immunoreactivity for STAT3 and STAT5A.

**Table 2:** Antibodies, instrument used, and antibody detection method were used in IHC

Antibodies	Antibody Clone	Catalog Number	Vendor	Dilution	Antigen Retrieval Method	Antibody Incubation Time	Pretreatment	Instrument	Detection System
**STAT3 Rabbit Monoclonal**	STAT3-Y705(D3A7)	9145	Cell Signaling, Beverly, MA	1:50	High pH	32 minutes	CC1 standard-high pH	Ventana Ultra	Ultraview Universal DAB Detection Kit
**STAT5A Rabbit Polyclonal**	STAT5A-Y694	T3794	Epitomics, Burlingame, CA	1:100	High pH	32 minutes	CC1 mild- high pH	Ventana Ultra	Optiview DAB IHC Detection Kit

The CC1 (High PH) for the Ventana Ultra - Tris Based Buffer.

The ER1 (Low PH) for the Bond III instrument - Citrate based pH 6.0 solution.

The ER2 (High PH) for the Bond III instrument - EDTA based pH 9.0 solution.

Abbreviations: CC: Cell Conditioner 1; DAB:3,3diaminobenzidine; IHC: Immunohistochemistry.

**Table 3:** STAT3 and STAT5A expression patterns in CRPC and BPH cases

Categories	Number of cases with focal positivity for STAT3	Number of cases with diffuse (>50% of the lesional cells) positivity for STAT5A	Number of STAT3 negative cases	Number of cases with focal positivity for STAT5A	Number of cases with diffuse (>50% of the lesional cells) positivity for STAT5A	Number of STAT5A negative cases
CRPC (n=15)	8 (Multifocal and strong)	6 (Diffuse and strong)	1	4 (Multifocal and strong)	8 (Diffuse and strong)	3
BPH (n=15)	7 (Focal and weak)	1(Diffuse and strong)	7	10 (Focal and weak)	None	5

Abbreviations: CRPC: Castration-resistant prostate cancer; BPH: Benign prostatic hyperplasia.

**Table 4:** Overall results of immnuohistochemical expression among the cases (expressed as percentage of cases immunoreactive to the specific antibody) among CRPC and BPH cases

Categories	STAT3 Positive Cases	STAT5A Positive Cases
CRPC (n=15)	14(93.3%)	12(80%)
BPH (n=15)	8(53.3%)	10(66.6%)

Abbreviations: CRPC: Castration-resistant prostate cancer; BPH: Benign prostatic hyperplasia.

**Table 5:** Immunohistochemical staining results for STAT3 and STAT5A represented in a semi-quantitative fashion as estimated percentage of tumor/lesional cells immunoreactive with the antibodies (0: no tumor/lesional cell staining, 1+: 1-25% of immunoreactive tumor/lesional cells, 2+: 26-50% of immunoreactive tumor/lesional cells, 3+: 51-100% of immunoreactive tumor/lesional cells) (expressed as number of cases)

Categories	STAT3 expression	STAT5A expression
CRPC (n=15)	3+ (Diffuse) staining, n=6 2+ (Multifocal) staining, n=8 1+ (Focal) staining, n=0 No staining, n=1	3+ (Diffuse) staining, n=8 2+ (Multifocal) staining, n=4 1+ (Focal) staining, n=0 No staining, n=3
BPH (n=15)	3+ (Diffuse) staining, n=1 2+ (Multifocal) staining, n=0 1+ (Focal) staining, n=7 No staining, n=7	3+ (Diffuse) staining, n=0 2+ (Multifocal) staining, n=0 1+ (Focal) staining, n=10 No staining, n=5

Abbreviations: CRPC: Castration-resistant prostate cancer; BPH: Benign prostatic hyperplasia.

### Increases in nuclear STAT3 and STAT5A staining in CRPC are statistically significant

CRPC cases showed significantly higher STAT3 nuclear positivity than BPH cases (Chi-square test, p=0.01). No significant differences were observed in focal and weak STAT5A expression between CRPC and BPH groups (Chi-square test, p=0.4). However, multifocal or diffuse and strong STAT3 (Chi-square test, p<0.0001) and STAT5A (Chi-square test, p<0.0001) expression was significantly higher in CRPC group than BPH group.

### CRPC cells express the low levels of STAT3 and STAT5A transcripts

**Figure 4:**
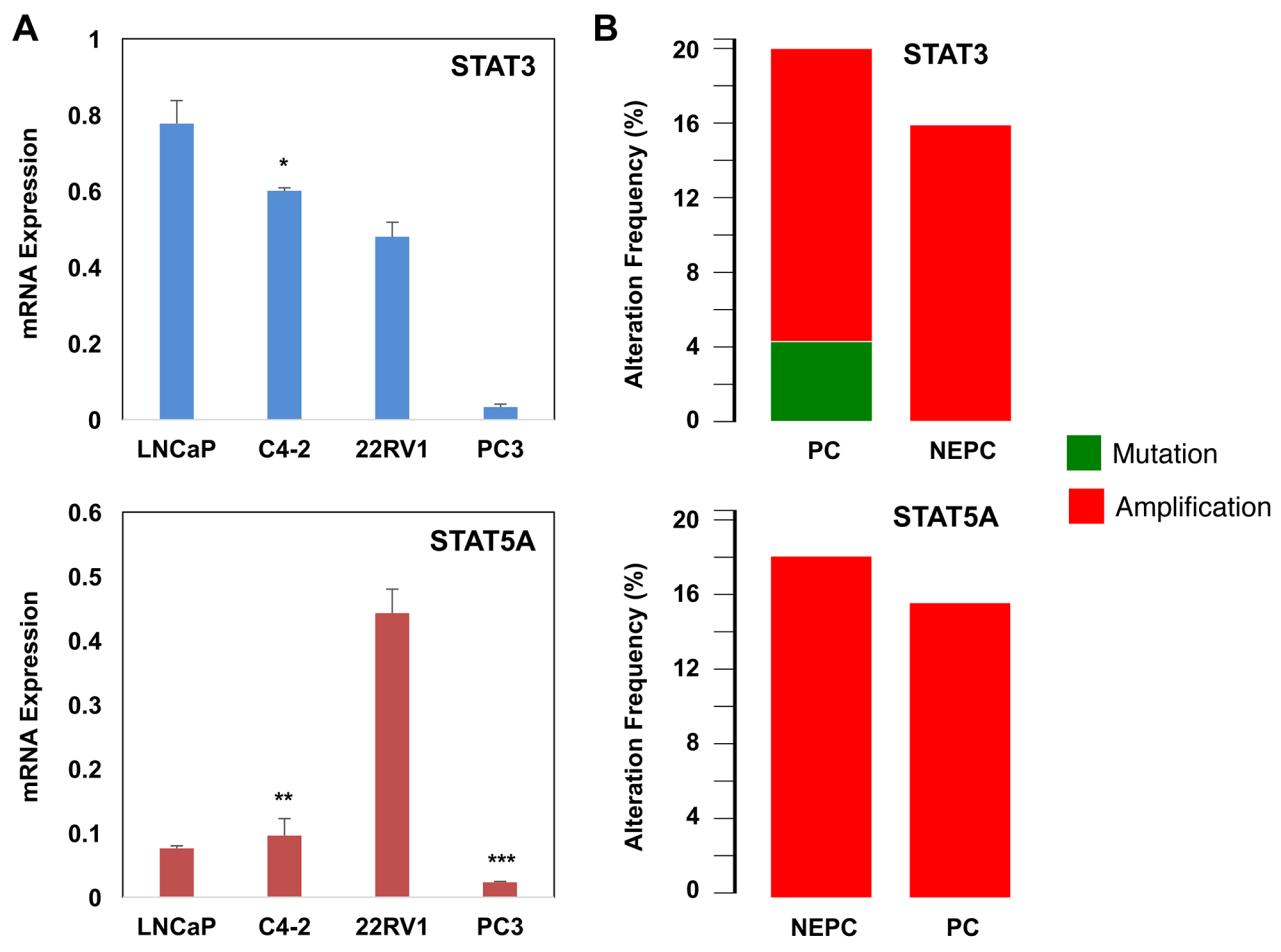
Alterations of STAT3/5A mRNA and genes in PC **(A**) Quantitative RT-PCR analysis of STAT3 and STAT5A transcripts in LNCaP, C4-2, 22Rv1, and PC3 cells. *p<0.0002, **p>0.05 ***p<0.002. 24h after cell seeding, total RNA was isolated from cells that were grown in serum-fed conditions. **(B)** Alterations of the STAT3 and STAT5A genes in metastatic CRPC clinical cases. The data were accessed through the www.cbioportal.org online platform. Data (±SD) are representation of two independent experiments in triplicates.

To complement the above clinical data, we performed a quantitative PCR analysis of STAT3 and STAT5A transcripts in PC cell lines LNCaP, C4-2, 22Rv1, and PC3. LNCaP is the AR-positive and castration-sensitive PC cell line [43]. C4-2 is the castration-resistant subline of LNCaP [44]. The AR-negative PC3 cell line is a bone metastatic model of human PC [45]. 22Rv1 is the castration-resistant subline of CW22R cells, and it was derived from human PC xenografts via serial circulation in castrated-mice [46]. The results of this analysis revealed that PC cells expressed varying levels of STAT3 and STAT5A transcripts (Figure 4A). LNCaP cells expressed the highest levels of STAT3 mRNA compared with C4-2, 22Rv1, and PC3. The levels of STAT3 mRNA were significantly (p<0.0002) lower in C4-2 and 22Rv1 than LNCaP cells. Although levels of STAT5A were slightly higher in C4-2 than LNCaP cells, the differences were not statistically (p>0.05) significant (Figure 4A). Surprisingly, however, 22Rv1 cells expressed the highest levels of STAT5A transcript relative to those of LNCaP, C4-2 and PC3. Unexpectedly, metastatic PC3 cells expressed the lowest levels of STAT3 and STAT5A transcripts compared with LNCaP, C4-2, and 22Rv1 (Figure 4A). In addition, we analyzed the PC public data set, which can be accessed through the www.cbioportal.org online platform, to see if metastatic CRPC cases exhibit altered patterns of the STAT3 and STAT5A genes. The results of our analysis showed that the STAT3 and STAT5A genes were amplified in up to 20% of metastatic CRPC clinical cases with the NE phenotype (Figure 4B). Here, we want to clarify that NE cells lack AR [40], and evidence suggested that the NE differentiation could be increased after ADT and in CRPC [15, 47].

### CRPC cells confer resistance to pimozide

**Figure 5:**
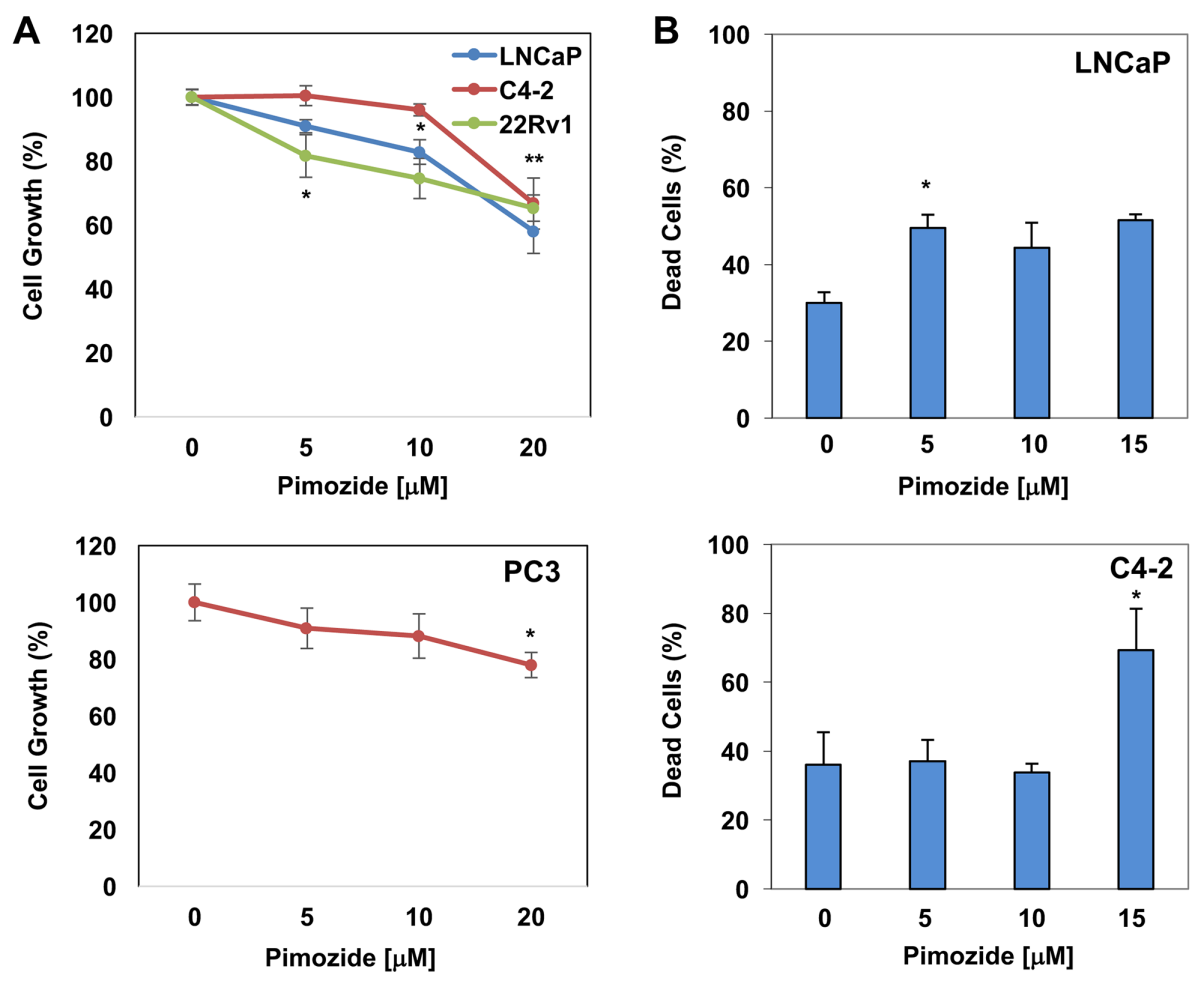
The effects of pimozide on PC cells growth and apoptosis *in vitro* **(A**) The dose-dependent effects of pimozide on LNCaP (*p<0.001), C4-2 (*p>0.06; **p<0.002), 22Rv1 (*p<0.02), and PC3 (*p>0.15; **p<0.002) cell growth. **(B**) Dose-dependent effects of pimozide on LNCaP (*p<0.01) and C4-2 (*p<0.01) apoptosis. Cell proliferation by CCK-8 and apoptosis by PI staining were analyzed at 48h post pimozide treatment. Data (±SD) are representation of two independent experiments in triplicates.

Pimozide was identified as an indirect inhibitor of STAT5 signaling and demonstrated to decrease the survival of chronic myelogenous leukemia cells that were resistant to the kinase inhibitors [39]. In that study, pimozide was demonstrated to inhibit STAT5 tyrosine phosphorylation, which coincided with inactivation of STAT5-dependent transcription [39]. To determine the effects of pimozide on PC cell growth and apoptosis, LNCaP, C4-2, and 22Rv1, and PC3 cells were exposed to varying doses of pimozide in cultures. The results showed that pimozide inhibited the growth of LNCaP, C4-2, 22Rv1, and PC3 cells in a dose-dependent manner (Figure 5A). Relative to mock control, 5 μM of pimozide was sufficient to suppress the growth of 22Rv1 cells significantly (p<0.02), whereas 10 μM of pimozide for LNCaP and 20 μM of pimozide for C4-2 and PC3 cells were required for a significant growth inhibition (p<0.002) under the same experimental conditions. In addition, we demonstrated that pimozide induced apoptosis in LNCaP and C4-2 cells in a dose-dependent manner (Figure 5B). We noted, however, that although 5 μM of pimozide was sufficient to significantly induce apoptosis in LNCaP cells (p<0.002), 20 μM of pimozide was necessary to achieve similar levels of apoptosis in C4-2 cells (p<0.007). Taken together, these observations indicate that CRPC cells C4-2 and PC3 confer resistance to pimozide, likely due to the low levels of STAT3/5A expression.

## DISCUSSION

Current therapeutic modalities available for patients with advanced PC include active surveillance, surgery [48], radiation therapy [48, 49], hormonal therapy [14, 18, 50], chemotherapy [18, 51, 52], and immunotherapy [53]. The clinical management of patients with PC depends on age, Gleason grade, prognostic grade group, overall health status of the patient, and the tumor stage. Currently, the mortality rate for recurrent PC is high, likely due to the lack of effective treatment. Therefore, effective pharmacotherapies designed to specifically target this lethal disease are much needed. Nevertheless, a better understanding of the cellular and molecular basis of CRPC is crucial for the identification of novel therapeutics. Evidence indicates the critical role of STAT3 and STAT5 in PC progression and metastasis. For example, activation of STAT3 and STAT5 has been implicated in treatment-naïve and advanced PC [30–38]. Don-Doncow *et al.* studied the protein and gene expression of STAT3 and IL6R in metastases from patients who died from CRPC [31]. The results of this study showed that 95% of metastatic cases expressed STAT3 and IL6R, as demonstrated by IHC. Interestingly, bone metastases showed significantly higher expression for both proteins compared to lymph node and visceral metastases. Similarly, STAT3 mRNA levels were significantly higher in bones than in lymph node and visceral metastases, whereas there was no significant difference in IL6R mRNA expression. In addition, using immunoblotting, immunofluorescence staining, and RT-PCR analysis, a study by Cocchiola *et al.* demonstrated that compared with the matched normal sections, prostate tumors with low Gleason scores (i.e. 6-7) exhibited a nuclear expression of pY705-STAT3 protein, whereas prostate tumors with high Gleason cases (i.e. 8–9) showed a cytoplasmic presence of pY705-STAT3 [30]. Likewise, evaluation of STAT3 protein in PC tissues by Western blotting showed a constitutively activated STAT3 (pY705) in all tumor grades compared to the matched normal sections.

Moreover, Singh *et al.* assessed on the expression and localization of STAT proteins by IHC in 150 formalin-fixed, paraffin-embedded human PC tissues with different Gleason scores [36]. A significantly strong STAT3 expression was seen in 68% PC cases as compared to only 12% of BPH cases (p<0.001). Of the cases with STAT3 expression, majority were of higher Gleason score (>7; 75%). 54% of the cases exhibited strong immunoreactivity for STAT5 as compared to only 13% of BPH controls (p<0.001), and 64% of the cases had a higher Gleason score (>7). A consistently intense nuclear staining of STAT proteins with high Gleason scores (>7) as compared to low scores (less than and equal to 7) was observed. Han *et al.* showed the usefulness of phosphorylated-STAT3 in detecting PC from negative biopsies [34]. STAT3 staining intensities in all samples (initial negative biopsies, cancer positive cores, and other negative cores from the same batch biopsies) of cancer patients was significantly higher than that of benign patients (p<0.001), with a high sensitivity (80.8%) and specificity (76.3%). Furthermore, Tam *et al.* studied the expression levels STAT pathway in the transition from a hormone-sensitive to hormone-refractory PC by assessing the expression levels of these IL-6R, JAK1, STAT3, pSTAT3 (Y705), and pSTAT3-Ser727 (S727) by IHC in 50 matched hormone-sensitive and hormone-refractory tumors pairs. An increase in expression of cytoplasmic IL-6 receptor, with the development of hormone-refractory PC was seen to be associated with reduced time to relapse, while an increase in expression of cytoplasmic pSTAT3 (Y705) was associated with reduced patient survival. In addition, those patients with high expression of cytoplasmic pSTAT3 (Y705) in their tumors had significantly shorter time to death from biochemical relapse and overall survival in comparison to those patients with low expression of cytoplasmic pY705-STAT3. Haddad *et al.* demonstrated amplification at the STAT5A/B gene locus in a significant fraction of clinical PC specimens [33]. The STAT5A/B gene amplification was more frequently found in PC with high histologic grades and in CRPC with distant metastases. Thus, the works presented herein suggest that targeting of STAT3 and STAT5 could in practice be of a therapeutic significance.

Here, we demonstrated that the levels of activated, nuclear STAT3 and STAT5A proteins were significantly elevated in metastatic CRPC compared with BPH. We recognize that although a limited number of CRPC clinical cases were analyzed, the result of our study is consistent with the findings in literatures [30, 31, 33, 34, 36, 38]. In addition, we demonstrated that human PC cell lines express differential levels of STAT3 and STAT5A transcripts. In general, compared with the STAT3, the expression of STAT5A is significantly low in LNCaP, C4-2, and PC3 cells, with an exception of 22Rv1 cell line, which expressed the highest levels of STAT3 mRNA. Moreover, our study revealed that metastatic PC3 PC cells expressed the lowest levels of STAT3 and STAT5A transcripts relative to that of LNCaP, C4-2, and 22Rv1. Furthermore, our analysis of the PC public data showed that the STAT3 and STAT5A gene locus were amplified in up to 20% of metastatic CRPC with the NE phenotype. Additionally, our data showed that C4-2 and PC3 cells expressing the low levels of STAT3/5A conferred resistance to pimozide in comparison with LNCaP and 22Rv1 cells under the same experimental conditions. Taken together, our results suggest that STAT3 and STAT5A can be utilized as a predictive biomarker to evaluate the efficacy of therapies targeting the JAK/STAT pathway.

Currently, there is no effective pharmacotherapy that prolongs the survival of patients with metastatic CRPC [11, 53–55]. To discover additional targeted agents, identification of the target population, or the subset of patients with druggable molecular abnormalities with minimal adverse effect, is necessary. The evaluation of both STAT3 and STAT5A expression in conjunction with morphology is important in therapeutic decision-making for patients who can benefit from the STAT inhibitor in pharmacotherapy. Here, we demonstrated that activated, nuclear expressions of STAT3 and STAT5A proteins was significantly high in metastatic CRPC cases. Nuclear STAT3 and STAT5A/B protein levels are increased in high-grade PC, CRPC, and distant metastases, and the high levels of nuclear STAT3 and STAT5A/B expression can predict early disease recurrence and PC-specific death in clinical PC. Based on the available data and our observation, STAT proteins represent a therapeutic target protein for advanced PC. Also, quantitative *in situ* analysis and STAT mRNA expression show that STAT3 and STAT5 gene amplification was associated with increased protein expression in PC. Functional studies showed that increased STAT3 and STAT5A/B copy numbers conferred growth advantage in PC cells *in vitro* and as xenograft tumors *in vivo* [31, 33, 38]. However, it is hard to estimate whether all STAT3/5 transcripts can fully translate into STAT3/5 protein, which ultimately drives the biology. Therefore, although intratumoral antigenic heterogeneity exists, we suggest that analysis of STAT3/5 protein expression may be a better way of predicting a therapeutic response.

In conclusion, our results demonstrate that STAT3/5A protein expression may potentially serve as a predictive biomarker of responsiveness to therapies targeting metastatic CRPC with NE characteristics. The JAK/STAT and AR pathways are functionally synergistic in the neoplastic cells and may involve the progression of PC [56–58]. Thus, the inhibition of the JAK/STAT signaling alone or in combination with the AR may lead to a novel treatment modality for patients with advanced PC. Further studies with a large cohort of metastatic CRPC patients with clinical follow-up information are warranted to substantiate the current findings and to evaluate whether there is a direct and positive correlation between the levels of activated STAT3/5A protein and induction of growth suppression and apoptosis in PC cell and animal models by pimozide. In addition, pimozide is a potent inhibitor of dopamine receptors (DRDs), particularly DRD2. Evidence suggests that activation of DRDs is linked to cancer with poor prognosis [59–63]. Therefore, future investigations warrant to establish whether the DRD and the JAK/STAT pathways mechanistically and functionally interact with each other to contribute to aggressive PC and that pimozide may disrupt the interactions, by which it suppresses PC cell growth.

## MATERIALS AND METHODS

### Tissue procurement

The Anatomic Pathology database was searched for CRPC cases from January 2008 to August 2011. All human subject research was conducted according to a protocol (#Pro00027882) approved by the Institutional Review Board (IRB). Fifteen cases of benign prostatic hyperplasia (BPH) were also included in this study as a control group. Demographics and related clinical details were recorded in each case. The clinical parameters including surgery, androgen ablation, chemotherapy, radiation therapy, androgen resistance, and metastasis were recorded. The hematoxylin and eosin (H&E) stained and relevant immunohistochemistry slides were reviewed to identify cases pertinent to this study.

### Immunohistochemistry

Immunohistochemical (IHC) staining of STAT3 and STAT5A proteins was performed on four-micrometer thick formalin-fixed and paraffin embedded (FFPE) tissues sections after confirmation of the diagnosis on H&E-stained sections. The automated immunostainer was used to stain STAT3 and STAT5A proteins in FFPE sections (Ventana Benchmark Ultra System, Roche). Table 1 summarizes the antibody clones, antibody dilutions, commercial vendors, antigen retrieval methods, and incubation time for the primary antibodies, and the localization techniques utilized in this study. Appropriate positive and negative controls were performed with each antibody. Positive staining of STAT3 and STAT5A protein was defined as a nuclear brown staining pattern that can be easily observed at low-power magnification (<×40) in the neoplastic cells in cancer cases and epithelial cells lining of the prostatic glands in BPH cases. Scant fine granular background staining of cells, which cannot be seen at low-power magnification (<×40) or no staining at all, was considered negative. The status of the immune markers was assessed without knowledge of the Gleason patterns and other clinical parameters of the cases. The staining results were recorded in semi-quantitative fashion as an estimated percentage of immune reactive tumor cells (in cancer cases) and benign prostate tissue (in BPH cases). 0: no staining, 1+ (focal), <25% of immunoreactive tumor or benign glandular epithelium; 2+ (multifocal), 26% to 50% of immunoreactive tumor or benign glandular epithelium; 3+ (diffuse): 51% to 100% of immunoreactive tumor or benign glandular epithelium. The staining intensity was recorded as weak, moderate, and strong.

### Cell lines and RNA isolation

PC cell lines LNCaP, C4-2, 22Rv1 and PC3 were grown in RPMI 1640 cell culture medium at 37°C in 5% CO_2_ incubator. The culture medium was supplemented with 10% fetal bovine serum (FBS) and 1% penicillin and streptomycin antibiotics (Thermo-Fisher Scientific). Total RNA from LNCaP, C4-2, 22Rv1, and PC3 cells that were grown at 70-80% confluency was isolated using RNA isolation kit according to manufacturer’s instruction (Thermo-Fisher Scientific).

### Quantitative RT-PCR

Levels of STAT3 and STAT5A transcripts were analyzed by reverse transcriptase-quantitative polymerase chain reaction (RT-qPCR) using GoTag® 1-step RT-qPCR system (Promega). Primer pairs are for STAT3 (Fv: 5’-GGA GCA GAG ATG TGG GAA TG-3’; Rv: 5’-GTG ATA CAC CTC GGT CTC AAA G-3’) and for STAT5A (Fv: 5’-CGG TTT GAG TGA GGG TTT CT-3’; Rv: 5’- GTG GGC AAC AGC ATC ATA GA-3’). Ribosomal 18S RNA was used as an internal control in RT-qPCR reactions to normalize the expression of STAT3 and STAT5A transcripts.

### Cell growth and apoptosis assays

LNCaP, C4-2, 22Rv1, and PC3 cells were seeded in 96 well plates (3x10^3^ cells/well) in serum-fed growth conditions 24h before exposed to various concentrations (0, 5, 10, and 20 μM) of pimozide, an indirect inhibitor of STAT5 activity/phosphorylation. Pimozide was obtained from Sigma-Aldrich. Pimozide was prepared in DMSO in 20 (mM) stock concentration and stored in -20 ^o^C. At 48h post treatment, cell proliferation or cell growth was assayed using a CellTiter 96 AQueous system (Promega) [64] or cell counting kit-8 (CCK-8) (Dojindo Molecular Technologies) in accordance with the manufacturer’s instruction. In a separate experiment and under the same growth and treatment conditions above, apoptosis induced by pimozide treatment was evaluated by Flow Cytometry using propidium iodide (PI) that stains apoptotic cells [65].

### Statistical analysis

Statistical comparison between the expression of STAT3 and STAT5A was studied among both CRPC and BPH groups. In addition, comparisons were made between the patterns of expression (semi-quantitative estimation of staining) for both groups for each marker. Important clinical parameters including age of the patient, Gleason score, and metastasis were compared with STAT3 and STAT5A expression in tumor groups. Pearson Chi-square test, Fischer’s exact test, and Student t-test (2-tailed) were used. Student *t*-test (2-tailed) was used to determine the significance between the two groups. A p-value of ≤ 0.05 was considered statistically significant.

## Abbreviations

PC: Prostate cancer
CRPC: Castration-resistant prostate cancer
AR: Androgen receptor
BPH: Benign prostatic hyperplasia
IRB: Institutional review board
IHC: Immunohistochemistry
STAT: Signal transducer and activator of transcription
JAK: Janus tyrosine kinase
FFPE: formalin-fixed and paraffin embedded
RT-qPCR: Reverse transcriptase-quantitative polymerase chain reaction
Fv: Forward
Rv: Reverse
PI: Propidium iodide
